# Inhibition of PERK Signaling Prevents Against Glucocorticoid-induced Endotheliocyte Apoptosis and Osteonecrosis of the Femoral Head

**DOI:** 10.7150/ijbs.35256

**Published:** 2020-01-01

**Authors:** Yanchun Gao, Hongyi Zhu, Qiyang Wang, Yong Feng, Changqing Zhang

**Affiliations:** Department of Orthopaedic Surgery, Shanghai Jiao Tong University Affiliated Sixth People's Hospital, 600 Yishan Road, Shanghai 200233, China

**Keywords:** glucocorticoid, osteonecrosis of femoral head, endoplasmic-reticulum stress, PERK signaling, endotheliocyte, apoptosis

## Abstract

Vascular injury is considered an important pathological process during glucocorticoid (GC)-induced osteonecrosis of the femoral head (ONFH). In this study, we tried to investigate whether the endoplasmic reticulum (ER) stress is triggered in the GC-induced endotheliocyte (EC) apoptosis and ONFH. The results showed that a GC upregulated the expression of ER stress-related proteins, and PERK-CHOP signaling played an important role and induced EC apoptosis. The inhibition of PERK by GSK2656157 significantly decreased the GC-induced EC apoptosis *in vitro* and *in vivo*, thus protecting a rat model from vascular injury and significantly preventing GC-induced ONFH.

## Introduction

Glucocorticoids (GCs) are widely used against a variety of diseases and are some of the most common causes of nontraumatic osteonecrosis of the femoral head (ONFH) 6, 15, 22, 26. It has been reported that osteonecrosis develops in 9-40% of patients receiving long-term GC therapy 35. Due to the lack of effective drugs for ONFH, patients often need surgical treatment. The long duration of this condition imposes persistent pain and an economic burden on the patients.

The adverse effects of GCs on bone are primarily due to direct actions, in particular, apoptosis of osteoblasts, osteoclasts, and endotheliocytes (ECs) 20, 42, 43. Many studies have suggested that the proapoptotic effect of GCs is cell type dependent 1, 13, 16, 34. Growing evidence shows that ECs are injured by a GC via induction of apoptosis and dysfunction during the pathological process of GC-induced ONFH 10, 20, 28, 46, 47. Thus, a better understanding of the mechanisms of action of GCs on ECs may lead to a better treatment option for ONFH.

The occurrence of endoplasmic reticulum (ER) stress-induced apoptosis has been proven in many diseases 7, 33. ER stress activates stress sensors, including protein kinase-like ER kinase (PERK), activating transcription factor 6 (ATF6), and inositol-requiring kinase 1 (IRE1), involved in the regulation of cell homeostasis. On the other hand, prolonged and unmitigated ER stress may cause apoptosis 2, 14, 33. Although ER stress correlates with various diseases, the role of ER stress in the pathogenesis of GC-induced ONFH remains unclear. A few studies have indicated that treatment with GCs leads to ER stress and results in various changes, including dysfunction and apoptosis of osteoblasts, osteocytes, and trabecular-meshwork cells 34, 44, 45. Nevertheless, few studies have focused on the GC-induced EC apoptosis mediated by the ER stress signaling pathway.

We were thus prompted to investigate whether ER stress is triggered during GC-induced EC apoptosis and ONFH, and, if so, whether GC-induced EC apoptosis and ONFH could be prevented by inhibition of the GC-induced ER stress signaling pathway.

## Materials and Methods

### *In Vitro* Experiments

#### Cell culture, treatment, and small interfering RNA (siRNA) transfection

The human EC line EAhy926 and human alveolar-bone-derived osteoblasts (OBs) were obtained from KeyGENE BIOTECH (Nanjing, China). The cell lines were cultured (37°C, 5% CO_2_) in DMEM supplemented with 10% of fetal bovine serum (Gibco, Grand Island, NY, USA), 100 U/mL penicillin, and 100 μg/mL streptomycin (Gibco). Human bone marrow stromal cells (BMSCs) were obtained from patients after amputation according to the method described by Kodama 21. Informed consent was obtained from all the patients. The BMSCs and OBs were also cultured (37°C, 5% CO_2_) in DMEM supplemented with 10% of FBS, 100 U/mL penicillin, and 100 μg/mL streptomycin (Gibco).

The siRNA experiment was conducted via delivery of plasmids. To construct siRNA expression vectors, the sequences were purchased from GenePharma (Shanghai, China). The siRNA sequences are listed in Appendix Table s1. ECs were transfected with siRNA against PERK, IRE1α, or ATF6 or scrambled siRNA (GenePharma, Shanghai, China). At 8 h after transfection, the transfection medium was replaced with a fresh culture medium, and the cells were cultured for 40 h before treatment with dexamethasone (DEX; Selleck, Houston, TX, USA). The transfection efficiency was >80%.

#### Annexin V/Propidium Iodide (PI) Fluorescence-Activated Cell Sorting (FACS) Analysis

Cells were analyzed for phosphatidylserine exposure by the annexin-V fluorescein isothiocyanate (FITC)/PI double-staining method according to the manufacturer's instructions (Dojindo Molecular Technologies, Inc. Gaithersburg, MD). Briefly, ECs were harvested by gentle trypsinization and then washed with PBS twice and resuspended in annexin-coupling buffer at a concentration of ~10^6^ cells/mL. A total of 100 μL of the cell suspension was incubated with 5 μL of the annexin V-FITC conjugate and 5 μL of a PI solution at room temperature for 15 min. A FACS machine was employed to evaluate the rate of apoptosis. Approximately 5,000 events were analyzed for apoptotic, necrotic, and live cells. All the experiments were repeated three times, and the results are expressed as a percentage of all the events in each experiment.

#### Western Blot Analysis

ECs were treated with DEX under different conditions. The cells were harvested and lysed with cell lysis buffer supplemented with protease and phosphatase inhibitor cocktails (Sigma-Aldrich, St. Louis, MO) on ice for 15 min. Protein samples were diluted 1:5 with protein loading buffer (Transgen Biotech, Beijing, China). A total of 30 µg of protein was subjected to SDS-PAGE after denaturation at 95°C for 5 min. The cell lysates were analyzed on a 10% gel (based on Tris-HCl buffer) under reducing conditions. After electrophoresis, the proteins were electrophoretically transferred to 0.22 μm polyvinylidene difluoride membranes (Merck, Darmstadt, Hesse, Germany) and blocked with 5% nonfat dry milk at 4°C overnight. The membranes were then incubated for 3 h at 37°C with anti-ATF6 (Thermo Fisher Scientific, Waltham, MA), anti-phosphorylated-IRE1α, (p-IRE1α; Abcam, Cambridge, MA), anti-PERK, anti-phosphorylated-PERK (p-PERK), anti-IRE1α, anti-CHOP, anti-BIP, anti-XBP1-s, anti-Caspase-3 (Casp3), anti-cleaved Caspase-3 (cCasp3), anti-β-Tubulin, or anti-GAPDH (Cell Signaling Technology, Danvers, MA) antibodies. The membranes were next immersed in a solution of a secondary antibody: an anti-rabbit or anti-mouse IgG antibody (Cell Signaling Technology) for 1 h at 37°C.

After three washes with Tris-buffered saline containing 0.1% of Tween 20, the membranes were added to an ECL substrate in a dark room for imaging on a FluorChem M Gel Documentation System (ProteinSimple, San Jose, CA, USA). The results were analyzed in densitometric analysis software Quantity One (Bio-Rad Laboratories, Inc., Hercules, CA, USA) β-Tubulin or GAPDH served as an internal reference.

### *In Vivo* Experiments

#### Establishment of the Osteonecrosis Model and Treatment

A short-term GC treatment model was set up as follows. The Sprague-Dawley (SD) rats were randomly and equally divided into the following three groups: 1 Control group (n = 10); 2 methylprednisolone (MPS) group (rats treated with MPS, n = 10); and 3 treatment group (osteonecrotic rats treated with MPS and PERK inhibitor GSK2656157, n = 10). In parallel, 0.2 mL of normal saline was intramuscularly injected into the rats in the control group. MPS (20 mg/[kg•d], Pfizer, New York NY) was intramuscularly injected once a day for 3 days.

The *in vivo* animal model of ONFH was constructed according to the description by Guo 11. The rats were randomly and equally subdivided into the control group (n = 10), MPS group (n = 10), and treatment group (n = 10).

According to a manufacturer's protocol, PERK inhibitor GSK2656157 (Selleck) was intragastrically administered (25 mg/kg) 8 h before every MPS injection in the treatment group.

None of the rats died before the scheduled euthanasia under 4% chloral hydrate anesthesia. Next, the femoral heads were collected for micro-computed tomography (CT) scanning, immunostaining, and immunohistochemical staining. All the experimental and animal care procedures were approved by the Animal Research Ethics Committee of Shanghai Sixth People's Hospital and were in compliance with the National Institutes of Health Guidelines for the Care and Use of Laboratory Animals.

#### Angiography and Micro-CT Scanning

Before euthanasia, the rats were anesthetized and successively perfused with 4% heparinized saline and Microfil (MV-112, Flow Tech, Carver, MA) according to the manufacturer's protocol. The bodies of the rats were stored at 4°C overnight, and then the bilateral femoral heads were collected for further experiments. The femoral heads were subjected to micro-CT scanning before and after decalcification in a 10% EDTA solution for 4 weeks. The micro-CD scanner was set to a resolution of 9 μm per pixel. The trabecular bone was segmented from the bone marrow and analyzed to determine trabecular thickness (Tb.Th), trabecular separation (Tb.Sp), trabecular bone pattern factor (Tb.pf), bone volume per tissue volume (BV/TV), and trabecular number (Tb.N). Three planes (coronal section, sagittal section, and transverse section) of the representative samples from each group were generated in the DataViewer software (Bruker Micro-CT). The total vessel volume was calculated by CTAn.

#### Immunofluorescent Staining

The femoral heads were sectioned at 5µm thickness in the coronal plane. The deparaffinized sections were processed by 0.25% trypsin antigen retrieval and were blocked with 10% FBS for 1 h at 37°C. The sections were incubated with anti-CD31 (1:200, Sigma-Aldrich) and anti-cleaved caspase 3 (cCasp3) (1:600, Cell Signaling Technology) antibodies at 4°C overnight (primary antibodies), and then the appropriate secondary antibodies (Servicebio, Wuhan, China) were applied for incubation at room temperature for 1 h. The nuclei were stained with 4′,6-diamidino-2-phenylindole (DAPI; Servicebio) for 5 min. Immunofluorescence photomicrographs were captured by means of a fluorescence microscope (Leica DMI6000B, Germany).

#### Immunohistochemical Staining

After 1 h 0.25% trypsin antigen retrieval and 1 h 10% FBS incubation at room temperature, the sections were incubated with the anti-cCasp3 (1:200, Cell Signaling Technology) primary antibody overnight at 4°C. After that, the sections were incubated with a biotinylated secondary antibody (Servicebio, Wuhan, China) according to the manufacturer's instructions. The sections were stained with a 3,3-diaminobenzidine precipitate and counterstained with hematoxylin. Photomicrographs were acquired using a LEICA DM 4000 (Leica Microsystems, Germany).

### Statistical Methods

Means of multiple groups were compared by one-way analysis of variance (ANOVA). Fisher's exact test was conducted to compare the incidence of the disease between two groups. The independent-sample *t* test was performed to compare means between two different groups. Statistical analysis was conducted in SPSS 20.0 software (IBM Corp., Armonk, NY, USA). Data with P values <0.05 were considered statistically significant.

## Results

### DEX Induces EC Apoptosis

**Figure 1:**
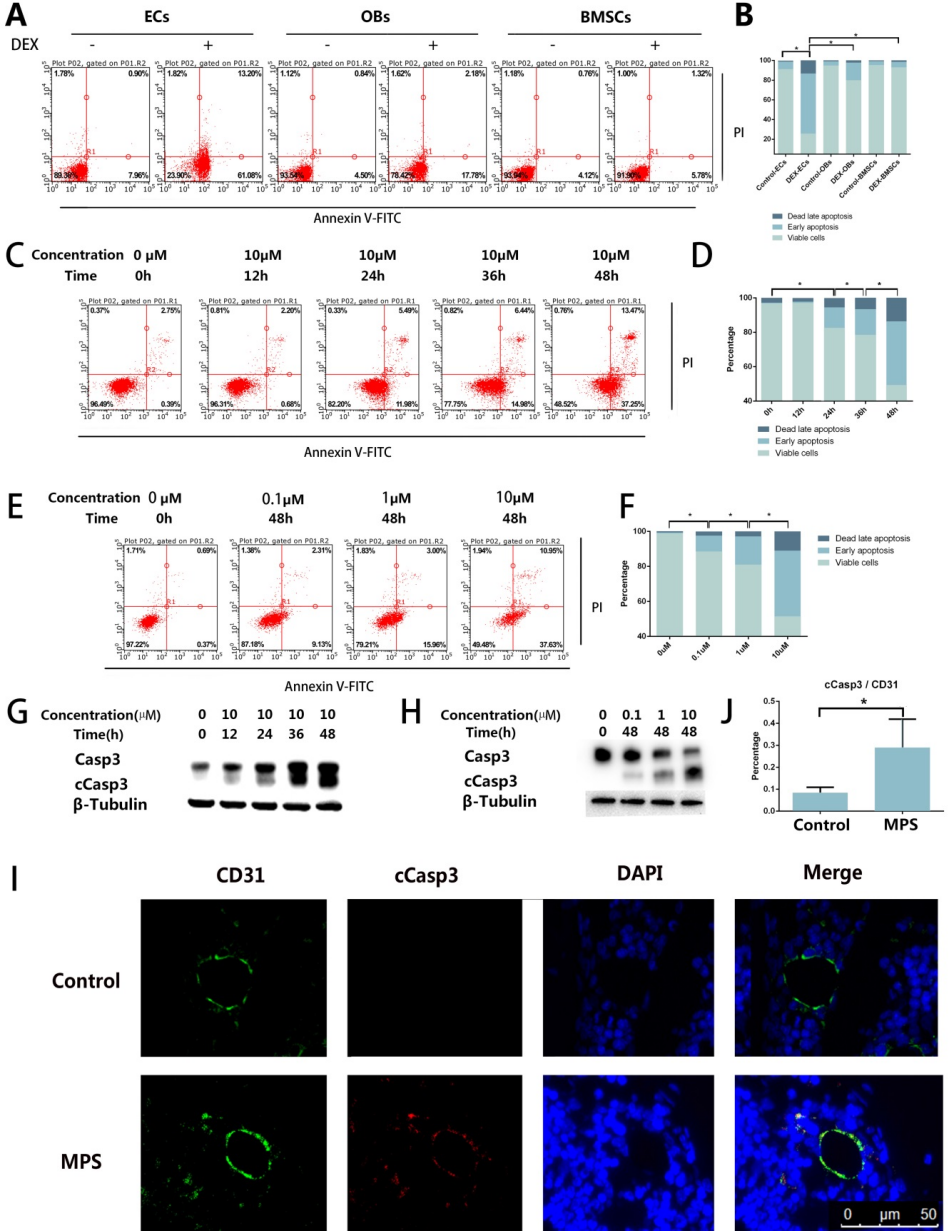
Apoptosis in the ECs in response to GC treatment. **A.** ECs, BMSCs, and MC3T3 were treated with DEX (10 µM) for 48 h. The FACS chart of Annexin V-FITC/PI staining is shown. **B.** The histogram of FACS results indicates the apoptotic status of different cells. **C.** ECs were treated with DEX (10 µM) for different periods (0-48 h). The FACS chart of Annexin V-FITC/PI staining is shown. **D.** The histogram of FACS results indicates the apoptotic status of the cells. **E.** Additional ECs were treated with different concentrations of DEX (0-10 µM) for 48 h. The results are presented as a FACS chart. **F.** The histogram of FACS results indicates the apoptotic status of the cells.** G, H.** ECs under the same treatment conditions presented in A and B were harvested from 6-well plates. The expression levels of Casp3 and β-tubulin are shown in a western blot. **I.** A representative image of cCasp3 immunohistochemical staining in the femoral head from a 3-day steroid-treated rat shows a pattern consistent with the shape of a microvessel. **J.** The histogram of immunofluorescence results indicates the percentage of apoptotic ECs in the femoral head. *p < 0.05.

The results of the flow cytometric analysis revealed DEX-induced apoptosis in ECs, BMSCs, and OBs. Compared with OBs and BMSCs, the ECs showed a stronger apoptotic tendency (Figure 1A, B). The flow cytometric analysis also revealed that the apoptosis of ECs increased with the increase of DEX stimulation time and concentration (Figure 1C-F). cCasp3, a representative activated Caspase involved in several types of cell death, was also detected here by western blotting, which supports the conclusions from the flow cytometric analysis (Figure 1G, H). To observe apoptosis *in vivo*, we performed cCasp3 and CD31 double-label immunofluorescence staining on rat femoral head sections. The results meant that short-term GC treatment induced apoptosis in the inner wall of blood vessels, whereas no apoptosis was found in other tissues of a femoral head (Figure 1I, J). We concluded that apoptosis occurs in the ECs in response to GC treatment, which revealed that the apoptosis was stronger in ECs than in other cells in bone tissue.

### DEX Induces ER Stress in ECs

**Figure 2:**
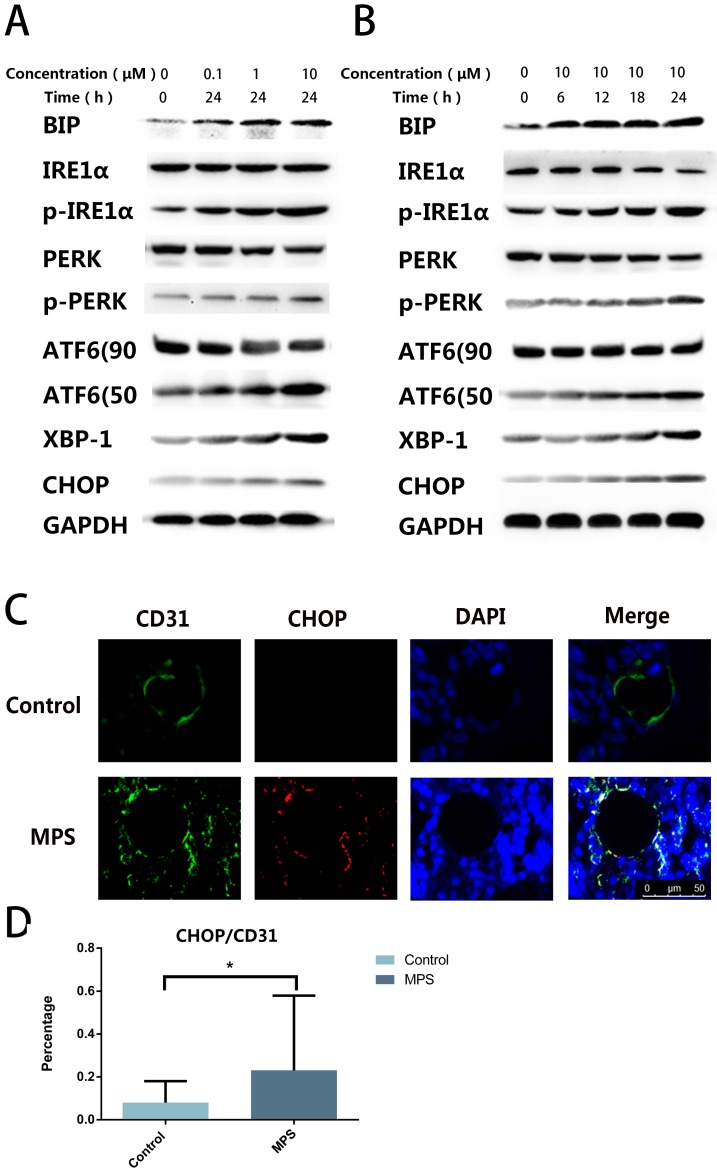
** A.** DEX Induces ER stress in ECs. ECs were treated with DEX at different concentrations (0-10 μm) for 24 h. ER stress-related proteins (PERK, p-PERK, IRE1α, p-IRE1α, ATF6, CHOP, XBP-1s, and BIP) were examined by western blotting. **B.** ER stress-related proteins were also observed among ECs treated with DEX for different periods (0-24 h, 10 μM). **C.** A representative image of CHOP immunohistochemical analysis in the femoral head from a 3-day steroid-treated rat shows a pattern consistent with the shape of a microvessel. **D.** The histogram of immunofluorescence results reveals the CHOP-positive percentage of ECs in the femoral head. *p < 0.05.

To find out the cause of apoptosis and to confirm the presence of ER stress, we quantified ER stress-related proteins including PERK, p-PERK, IRE1α, p-IRE1α, ATF6, CHOP, XBP-1s, and BIP. The western blot revealed that ER stress-related proteins were upregulated with the increase of stimulation time and concentration, and three classical signaling pathways were found to be activated simultaneously after DEX treatment (Figure 2A, B). The CHOP and CD31 double-label immunofluorescence staining indicated that CHOP expression in ECs increased after short-tern GC treatment (Figure 2C, D). These findings confirmed GC-induced ER stress in ECs.

### PERK Inhibition Prevents EC Apoptosis *In Vitro*

**Figure 3:**
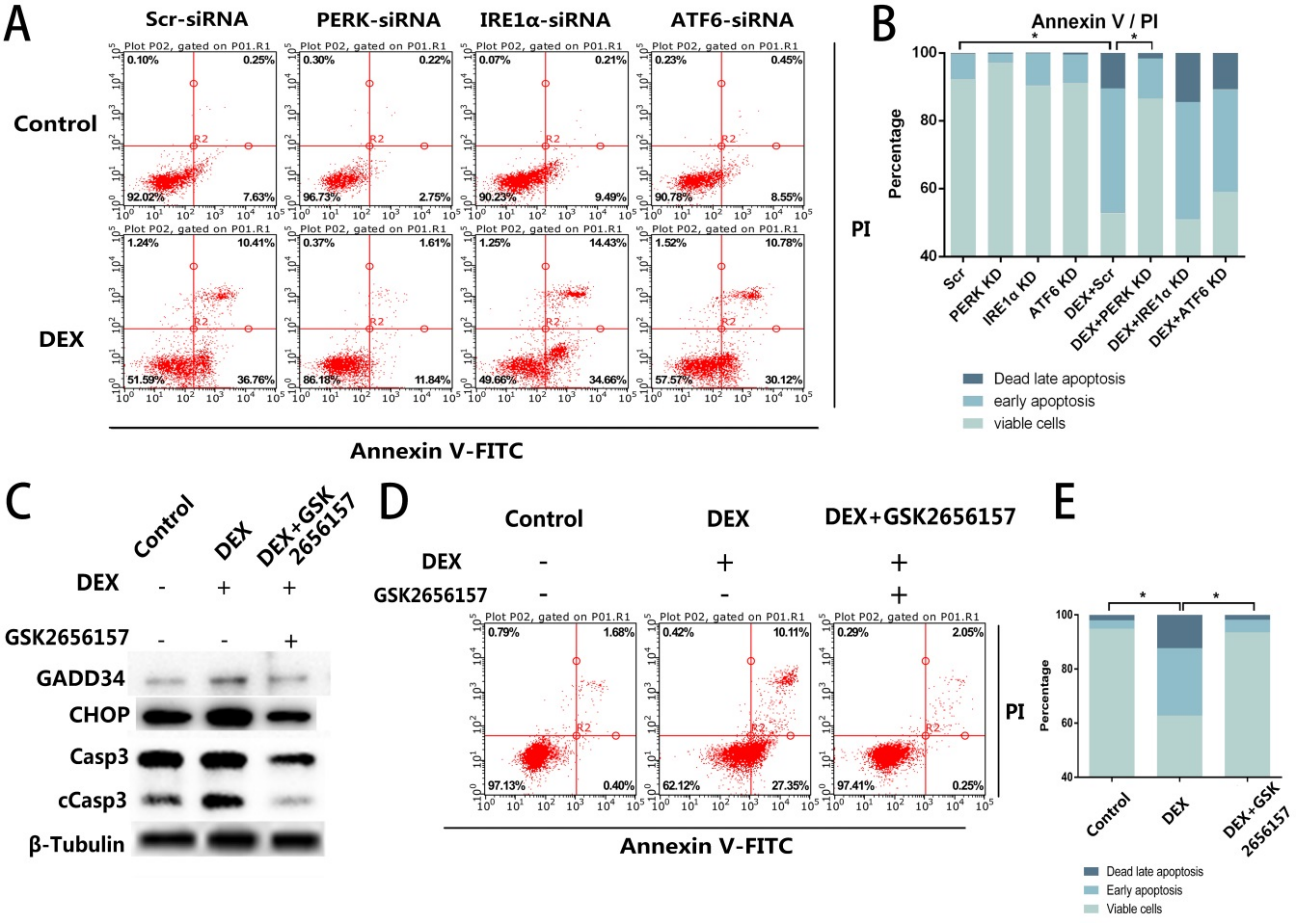
PERK inhibition by siRNA or PERK inhibitor GSK2656157 decreased the apoptosis of ECs *in vitro*. **A.** ECs were transfected with siRNAs to knockdown PERK, IRE1α, or ATF6, followed by treatment with vehicle or DEX (10 µM) for 48 h. The FACS charts of the Annexin V-FITC/PI staining are presented. **B.** The histogram indicates the apoptotic status of the cells. **C.** The ECs were treated under the same above-mentioned conditions, and the expression levels of Casp3 were examined by western blotting. **D.** The cells were pretreated with the PERK inhibitor GSK2656157 8 h before the DEX treatment (10 µM; 48 h). The FACS charts are shown. **E.** The histogram of the Annexin V-FITC/PI staining is shown. *p < 0.05.

Given that we observed the activation of all three ER stress-related downstream signaling pathways, further experiments were necessary to determine the specific pathway associated with the apoptosis. We blocked the three signaling pathways respectively by transfection of short interfering RNAs against PERK, IRE1α, or ATF6. The siRNA-treated ECs were then stimulated with DEX and assessed by flow cytometry. Compared with the control group, neither the IRE1α-deficient nor ATF6-deficient ECs showed a decrease of apoptosis, whereas the inhibition of PERK significantly decreased the apoptosis after DEX treatment (Figure 3A, B).

After that, we identified a PERK inhibitor, GSK2656157, and pretreated the cells with the inhibitor 8 h before the DEX treatment. Downstream of PERK signaling pathway. We quantified GADD34 and CHOP, which are downstream of the PERK signaling pathway and participate in apoptosis. The western blot showed that the expression of GADD34 and CHOP diminished after GSK2656157 treatment. Besides, the decreased expression of cCasp3 indicated the antiapoptotic effect of PERK inhibition (Figure 3C). The flow cytometric analysis also showed the decrease of apoptosis in the GSK2656157-pretreated ECs, indicating that the PERK inhibitor exerted a protective action against GC-induced apoptosis (Figure 3D, E).

### PERK Inhibition Prevents the GC-induced Vascular Damage *In Vivo*

**Figure 4:**
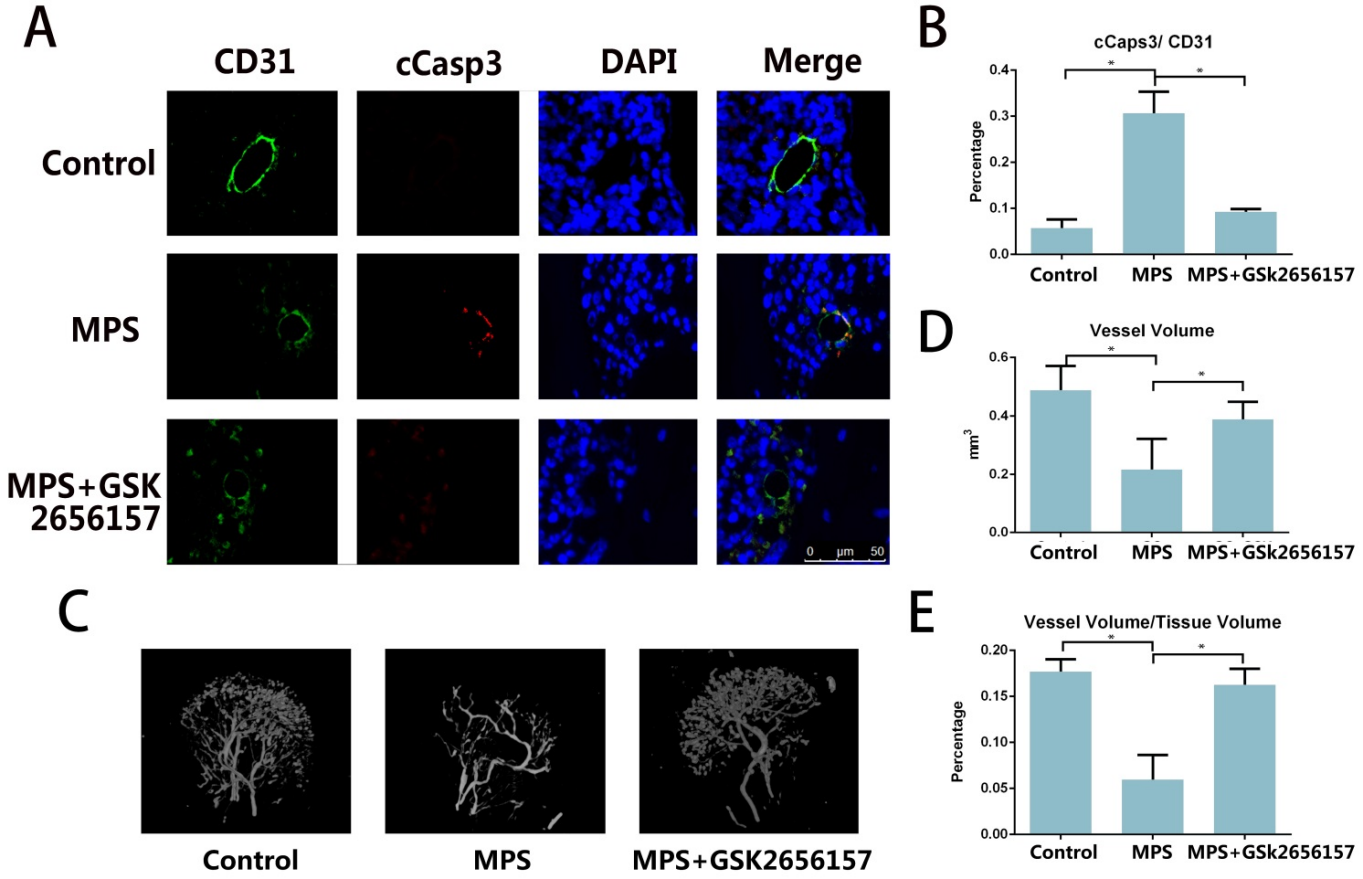
PERK inhibitor GSK2656157 prevented vascular damage in an ONFH model. **A.** Representative immunostaining of the femoral heads from the rats that received 3-day steroid injections shows the colocalization of CD31 and cCasp3 in the GC group, whereas the immunostaining of the femoral heads from both the control and treatment groups shows low expression levels of cCasp3. **B.** The histogram based on densitometric analysis of immunostaining from various groups (n = 10; * p < 0.05). **C.** A representative 3D reconstructed microangiographic image shows the general form of the intraosseous microvessels in the femoral head. **D, E.** Quantification of the total vessel volume (mm^3^) and the ratio of total vessel volume to total tissue volume in the femoral heads from the different groups are shown (n = 10). *p < 0.05

The immunofluorescent staining of CD31 and cCasp3 revealed that PERK inhibitor GSK26556157 reduced the EC apoptosis after short-term MPS treatment. The PERK inhibitor provided protection against GC-induced apoptosis, as verified by our findings *in vitro* (Figure 4A, B) The micro-CT analysis of the perfused vessels in the femoral heads also suggested that the long-term GC treatment injured the vessels in the femoral head. The PERK inhibitor GSK2656157 had a protective effect against GC-induced vascular injury (Figure 4C-E).

### PERK inhibitor GSK2656157 Prevents GC-induced ONFH *In Vivo*

**Figure 5:**
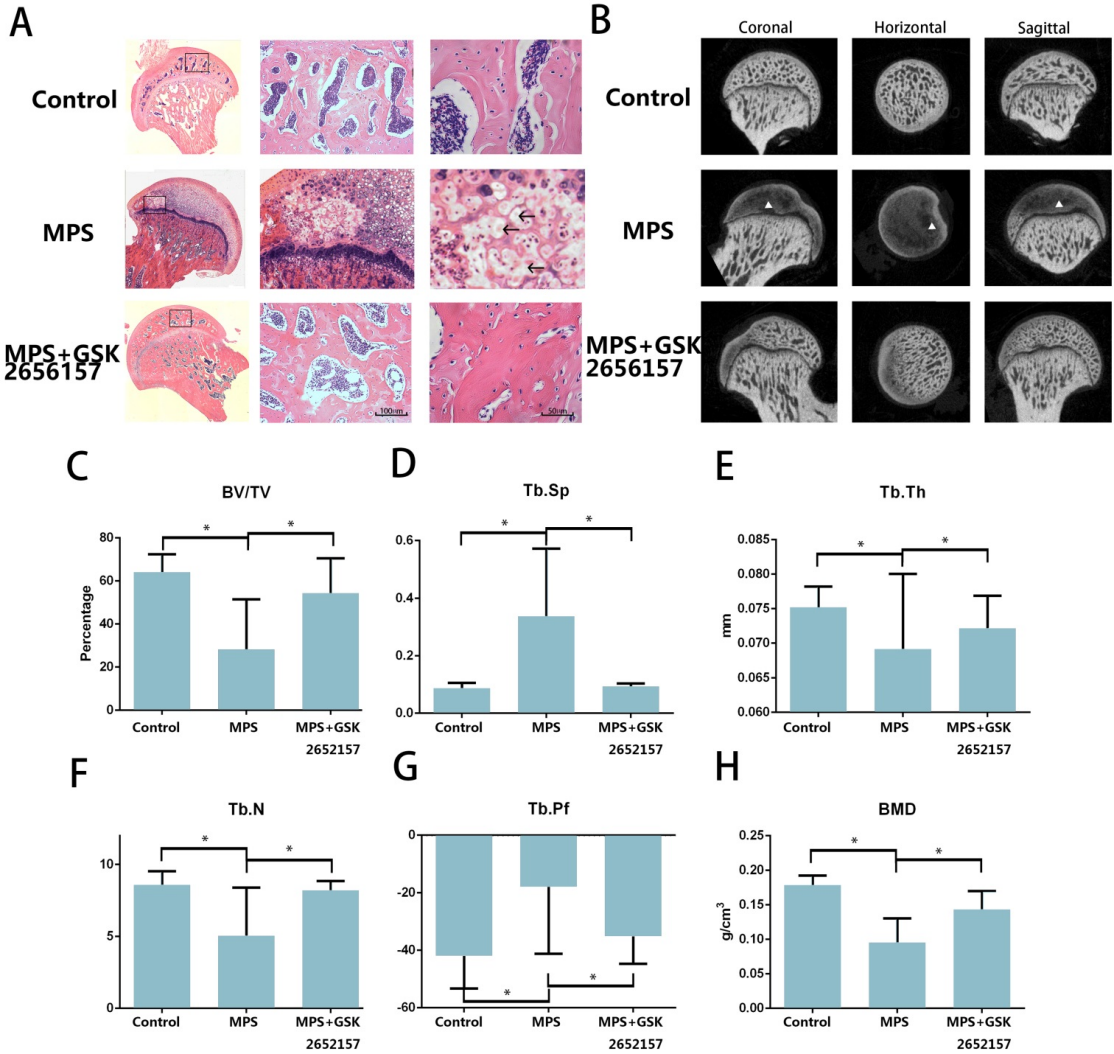
PERK inhibitor GSK2656157 protected the femoral head from osteonecrosis *in vivo*. **A.** The H&E staining of coronal sections of the representative femoral heads from each group. The arrows indicate the empty lacunae and hypertrophic fat cells, which indicate osteonecrosis. **B.** Representative images from the micro-CT analyses in the sagittal, horizontal, and coronal planes. The triangle indicates lower density changes in the subchondral area in the ONFH group. **C-H.** The histograms of the morphometric analysis show the BMD and bone parameters (BV/TV, Tb.Th, Tb.N, Tb.Pf, and Tb.Sp) of the upper outer subchondral bones of the femoral heads (n = 20). *p < 0.05

The results of an *in vitro* experiment revealed that 13 in 20 femoral heads (10 Sprague-Dawley rats) in the MPS group showed visible signs of osteonecrosis, while only one osteonecrotic femoral head was found in the treatment group (p < 0.001). Hematoxylin and eosin (H&E) staining revealed visible subchondral necrosis with fatty-tissue invasion in the subchondral bone trabecular area, whereas PERK inhibitor GSK2656157 successfully prevented ONFH in the treatment group (Figure 5A). The trabecular changes in the subchondral region of the femoral heads were detected by micro-CT (Figure ​5B). Besides, the micro-CT analysis yielded results on bone parameters, including Tb.Th, Tb.Sp, Tb.pf, and BV/TV. We noticed a significant preventive effect of GSK2656157 (Figure ​5C-G). The bone mineral density (BMD) of the rats in the model group was 0.076 g/cm^3^, which was significantly lower than that of the control group, whereas the PERK inhibitor significantly prevented the reduction in BMD (Figure ​5H). The results above led to the conclusion that PERK signaling blockage (by GSK2656157) may prevent ONFH.

## Discussion

The most important findings of this study are that ER stress is strongly involved in GC-induced EC apoptosis and ONFH. ECs are more sensitive to apoptosis than other cells in bone tissue when stimulated by GCs. PERK-CHOP signaling plays a critical part in this process. The PERK inhibitor GSK2656157 was demonstrated to be effective in preventing GC-induced vessel injury and ONFH *in vivo*, thereby further proving the decisive participation of PERK signaling in GC-induced ONFH. This study provides new insight into the inherent relation among EC apoptosis, vascular injury, and GC-induced ONFH. Namely, GCs cause ER stress in ECs and induce apoptosis, which leads to microvascular damage and eventually causes ONFH.

Apoptosis in bone is thought to be the key determinant of GC-induced ONFH 17, 18, 43. Some studies have mainly focused on the direct adverse effects of administered GCs on BMSCs, osteoblasts, and osteoclasts; GCs decrease the formation of both osteoblasts and osteoclasts and increase the apoptosis of osteoblasts while prolonging the lifespan of osteoclasts 12, 34, 36, 37, 43, 48. However, recent studies pointed out that apoptosis of ECs in ONFH are essential but ignored. One of the possible reasons is that the apoptotic effect of a GC is cell-type dependent 9, 46. Compared to other cells, ECs show a stronger and earlier apoptotic tendency in response to GC treatment. Furthermore, there is an even higher GC concentration in blood vessels than in other tissues *in vivo* 30, 38. It has been reported that during high-dose GC treatment (blood concentration of 15-100 µM, according to a GC treatment guide) 39, the vessels are more affected than any other tissues in the femoral head. This observation is consistent with earlier studies, which suggest that vessel injury is an initiating factor for the pathological processes of osteonecrosis 19, 25, 47, and that osteonecrosis is a series of secondary pathological changes in response to ischemia 4.

ER stress is known to activate three major signaling pathways, such as the PERK-ATF4 axis, ATF6 signal transduction, and IRE1a cascade 24, 33, 41. ER stress may act as a two-edged sword in such diseases as ONFH. The ER responds to stress by activating the unfolded protein response (UPR). If various UPR-induced mechanisms fail to alleviate ER stress, both the intrinsic and extrinsic pathways of apoptosis can get activated. When activated upon sensing ER stress, PERK oligomerizes and phosphorylates itself and the ubiquitous translation initiation factor eIF2α, thereby indirectly inactivating eIF2α and inhibiting mRNA translation. In this way, PERK helps reduce the flux of protein entering the ER to alleviate ER stress 40. It has been demonstrated that a dephosphorylation inhibitor of eIF2α can reduce ER stress 3, 25, 34. Nevertheless, strong or prolonged ER stress may break the homeostatic balance of UPR 32, 40. Subsequently, the PERK-CHOP signaling pathway is activated, inducing apoptosis 5, 8, 24, 31.

PERK is the major protein responsible for attenuation of mRNA translation under ER stress, preventing the influx of newly synthesized protein molecules into the already stressed ER compartment. The activation of eIF2α phosphorylation has been demonstrated to reduce the flux of protein into the ER and thus alleviate ER stress. Nonetheless, different levels of stimulation have been found to cause various ER stress responses. Prolonged or extra strong stimulation is thought to break the PERK-eIF2α regulatory mechanism, mediated by the activation of ATF4-CHOP signaling, which has a proapoptotic activity and is crucial for triggering apoptosis in response to ER stress 29, 31, 33, 40. The experiment on the relation between GC-induced ER stress and stimulus intensity (time/concentration) was under consideration. Because apoptosis in patients with steroid-induced ONFH has been widely reported, it is believed that long-term or large-dose GC treatment may directly cause this apoptosis 27.

More recently, a few studies described the participation of ER stress in ONFH. Sato AY et al. have reported that salubrinal or guanabenz, specific inhibitors of eIF2α dephosphorylation, block apoptosis of osteocytic MLO-Y4 and osteoblastic OB-6 cells, when this apoptosis is induced by either GC or ER stress inducers 34. Liu et al. have reported that ER stress is an important pathological outcome in the surgery-induced ONFH model, and salubrinal alleviates ONFH symptoms by enhancing angiogenesis and bone healing via suppression of ER stress 23. These two studies show that inhibition of eIF2α dephosphorylation reduces ER stress and is proven to be effective at launching the ER stress-related response 3. Furthermore, they focused on the direct effects of ONFH on osteoblasts and osteoclasts. In this study, we proved that a GC activates PERK-CHOP signaling, indicating that GC-induced ER stress breaks ER homeostasis and leads to apoptosis. Furthermore, we confirmed that ER stress is strongly involved in GC-induced EC apoptosis and ONFH. GSK2656157, an inhibitor of PERK phosphorylation, has been proven to be effective against ER stress-induced apoptosis 23. In our study, GSK2656157 blocked the whole downstream signaling of PERK and attenuated GC-induced cell apoptosis. GSK2656157 was demonstrated to be effective in the protection from steroid-induced vessel injury and from ONFH *in vivo*. These findings confirmed the decisive role of PERK signaling in ONFH.

Our study has several limitations. First, a CHOP knockout transgenic model was not constructed due to the lack of osteonecrotic murine models. Second, although the therapeutic effect is clear-cut, PERK inhibitor GSK2656157 is still not an approved drug and further clinical studies are required.

This work should improve the understanding of the relation between intraosseous microvascular injuries and steroid-induced ONFH. GC-induced ER stress was found to induce EC apoptosis, which triggers other secondary changes and finally causes osteonecrosis. Our experimental results also suggest a promising drug for the prevention of ONFH. PERK signaling, as a brand-new research direction for ONFH prevention, requires much lucubration in the future. Furthermore, a more in-depth study on ONFH may change the traditional paradigm of several bone diseases and could contribute to the discovery of a novel therapeutic method.

## Supplementary Material

Supplementary figures and tables.

## Abbreviations

ATF6: activating transcription factor 6
BIP: immunoglobulin heavy-chain-binding protein
BMSCs: bone marrow stromal cells
Casp3: caspase 3
CHOP: C/EBP-homologous protein
cCasp3: cleaved caspase 3
DEX: dexamethasone
EC: endotheliocyte
ER: endoplasmic reticulum
FACS: fluorescence-activated cell sorting
GC: glucocorticoid
IRE1: inositol-requiring kinase 1
OBs: osteoblasts
ONFH: osteonecrosis of the femoral head
PERK: protein kinase-like ER kinase
p-IRE1α: phosphorylated IRE1α
p-PERK: phosphorylated-PERK
XBP1s: X-box-binding protein 1 sliced
