# Inhibition of PERK Signaling Prevents Against Glucocorticoid-induced Endotheliocyte Apoptosis and Osteonecrosis of the Femoral Head: Erratum

**DOI:** 10.7150/ijbs.58918

**Published:** 2021-03-19

**Authors:** Yanchun Gao, Hongyi Zhu, Qiyang Wang, Yong Feng, Changqing Zhang

**Affiliations:** Department of Orthopaedic Surgery, Shanghai Jiao Tong University Affiliated Sixth People's Hospital, 600 Yishan Road, Shanghai 200233, China

In our paper 1, there were two errors due to the mishandling of Western Blot picture and Flow Cytometry data during the final preparation of manuscript. We regret that we did not detect these errors before publication. Here we showed the corrected Figure 2A.B and Figure 3D with side by side comparison of the previous figures. The data analyses and conclusion remain unchanged.

## Figures and Tables

**Figure 2:**
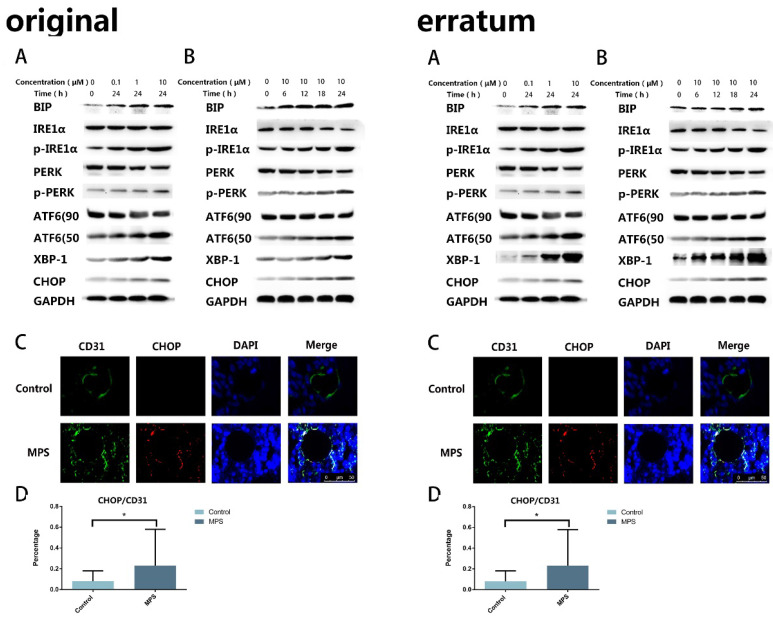
A.B

**Figure 3:**
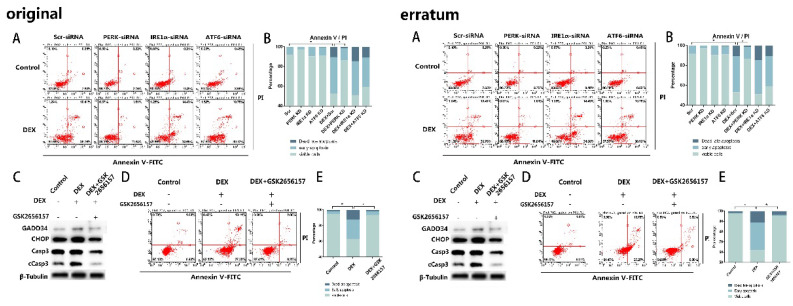
D
