# Supplementary material for: Inhibition of PERK Signaling Prevents Against Glucocorticoid-induced Endotheliocyte Apoptosis and Osteonecrosis of the Femoral Head
Source: Int J Biol Sci. 2020 Jan 1;16(4):543–52. doi: 10.7150/ijbs.35256 (PMC6990927; doi:10.7150/ijbs.35256)
Supplement: Supplementary file 1 — Supplementary figures and tables. [file ijbsv16p0543s1.zip › Appendix Table 1.docx]

Appendix Table 1

| Scrambled control | sense 5’-UUCUCCGAACGUGUCACGUTT-3 |
| --- | --- |
|  | antisense 5’-ACGUGACACGUUCGGAGAATT-3 |
| PERK | 5’-GUGGAAAGGUGAGGUAUAUTT-3 |
|  | antisense5’-AUAUACCUCACCUUUCCACTT-3 |
| IRE1α | sense 5’-GUCCCACUUUGUGUCCAAUTT-3 |
|  | antisense 5’-AUUGGACACAAAGUGGGACTT-3 |
| ATF6 | sense 5’-GUGGACUCUUAUUCUUCAATT-3 |
|  | antisense 5’-UUGAAGAAUAAGAGUCCACTT-3 |
