# Supplementary figures and images for: Inhibition of PERK Signaling Prevents Against Glucocorticoid-induced Endotheliocyte Apoptosis and Osteonecrosis of the Femoral Head
Source: Int J Biol Sci. 2020 Jan 1;16(4):543–52. doi: 10.7150/ijbs.35256 (PMC6990927; doi:10.7150/ijbs.35256)

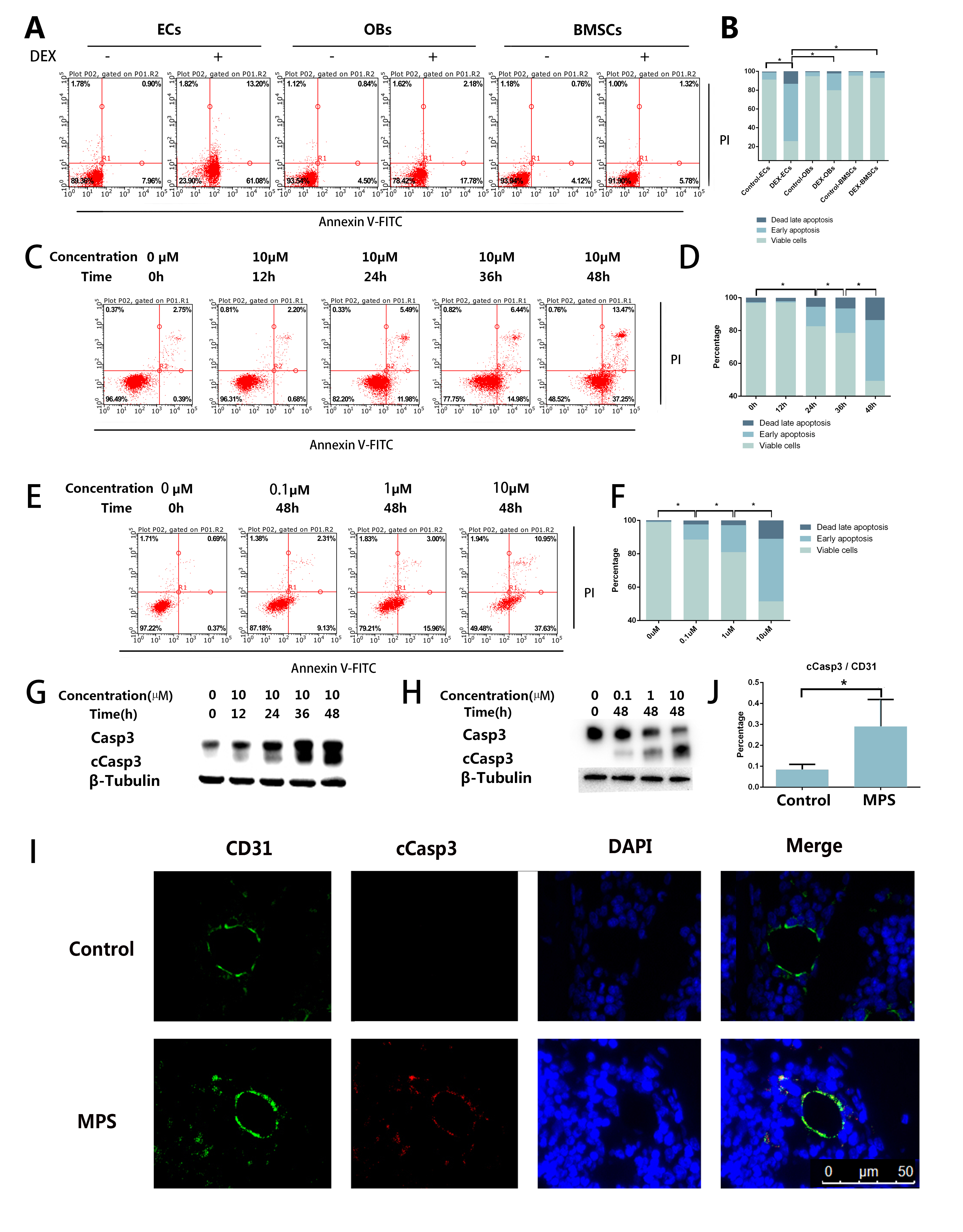

Supplement: Supplementary file 1 — Supplementary figures and tables. [file ijbsv16p0543s1.zip › Fig.1.jpg]

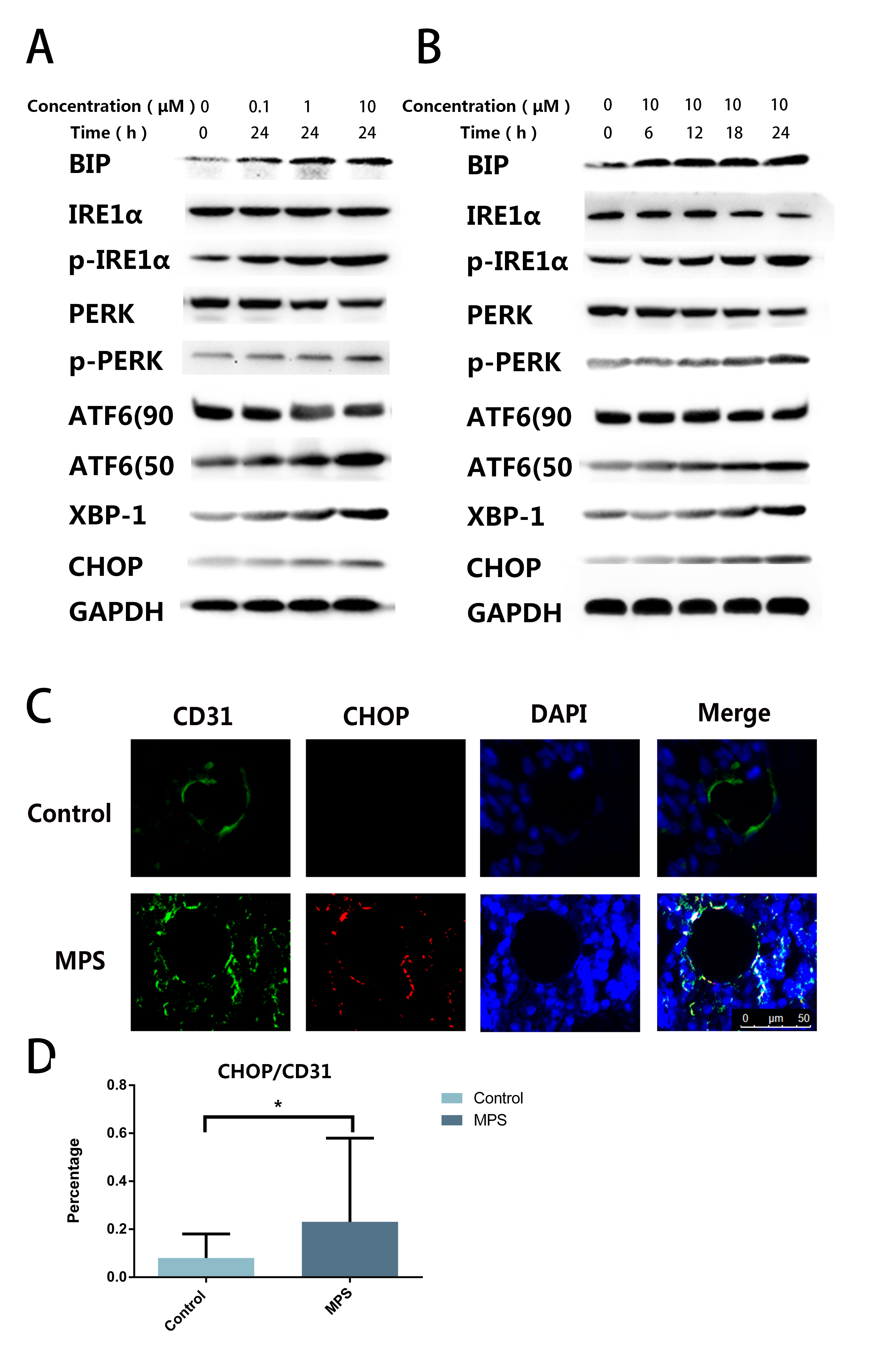

Supplement: Supplementary file 1 — Supplementary figures and tables. [file ijbsv16p0543s1.zip › Fig.2.jpg]

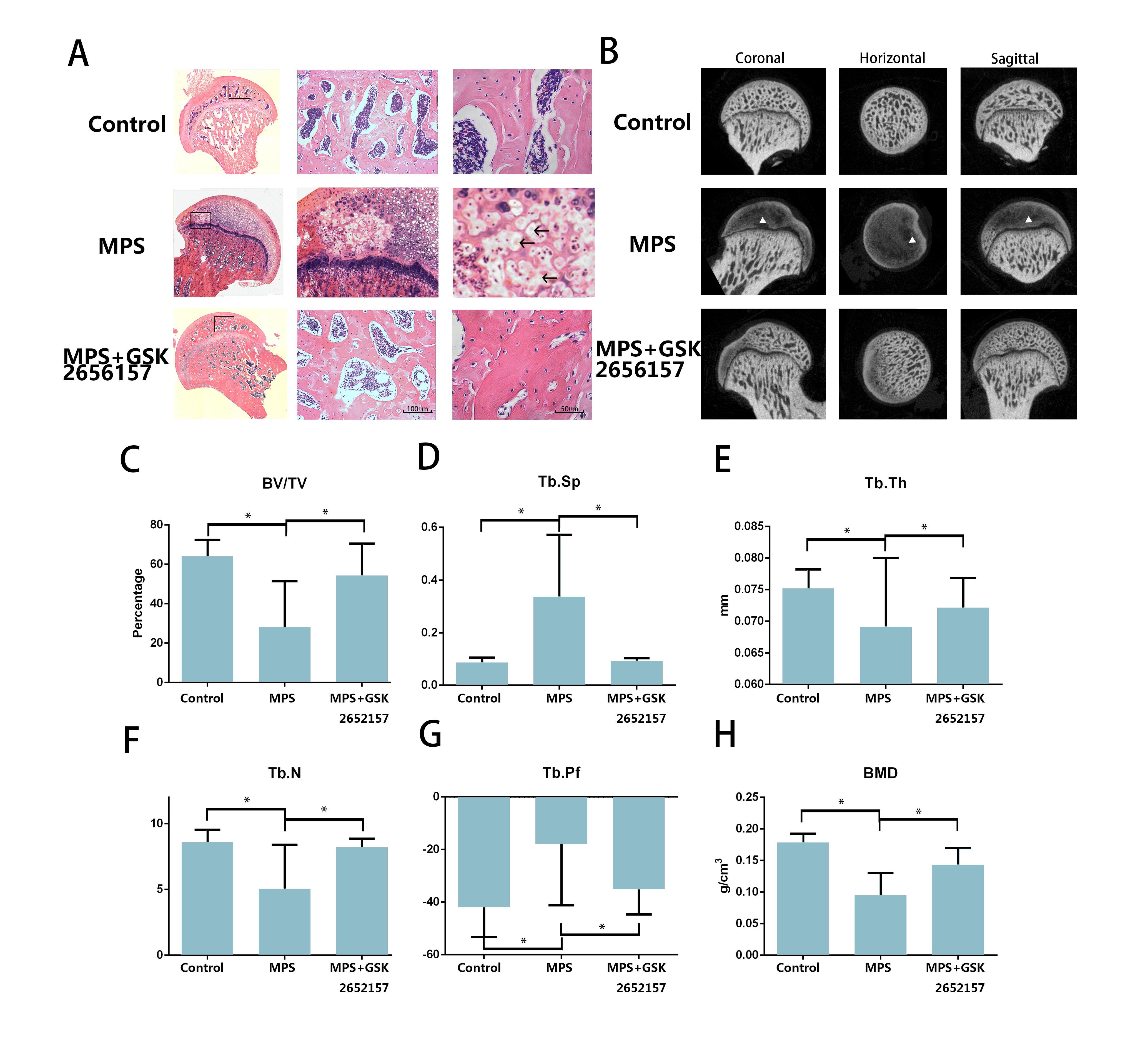

Supplement: Supplementary file 1 — Supplementary figures and tables. [file ijbsv16p0543s1.zip › Fig.5.jpg]
